# PARP1 Is Up-Regulated in Non-small Cell Lung Cancer Tissues in the Presence of the Cyanobacterial Toxin Microcystin

**DOI:** 10.3389/fmicb.2018.01757

**Published:** 2018-08-06

**Authors:** Patrick L. Apopa, Lisa Alley, Rosalind B. Penney, Konstantinos Arnaoutakis, Mathew A. Steliga, Susan Jeffus, Emine Bircan, Banu Gopalan, Jing Jin, Preecha Patumcharoenpol, Piroon Jenjaroenpun, Thidathip Wongsurawat, Nishi Shah, Gunnar Boysen, David Ussery, Intawat Nookaew, Pebbles Fagan, Gurkan Bebek, Mohammed S. Orloff

**Affiliations:** ^1^Department of Epidemiology, University of Arkansas for Medical Sciences, Little Rock, AR, United States; ^2^Department of Environmental and Occupational Health, University of Arkansas for Medical Sciences, Little Rock, AR, United States; ^3^Winthrop P. Rockefeller Cancer Institute, University of Arkansas for Medical Sciences, Little Rock, AR, United States; ^4^Department of Pathology, University of Arkansas for Medical Sciences, Little Rock, AR, United States; ^5^Cleveland Clinic, Cleveland, OH, United States; ^6^Department of Biomedical Informatics, University of Arkansas for Medical Sciences, Little Rock, AR, United States; ^7^College of Medicine, University of Arkansas for Medical Sciences, Little Rock, AR, United States; ^8^Department of Health Behavior and Health, University of Arkansas for Medical Sciences, Little Rock, AR, United States; ^9^Department of Electrical Engineering and Computer Science, Case Western Reserve University, Cleveland, OH, United States; ^10^Center for Proteomics and Bioinformatics, Case Western Reserve University, Cleveland, OH, United States; ^11^Department of Nutrition, Case Western Reserve University, Cleveland, OH, United States

**Keywords:** microbiome, NSCLC, lung, adenocarcinoma, inflammation, *CD36*, 16S rRNA, *Cyanobacteria*

## Abstract

Non-small cell lung cancer (NSCLC) is the major form of lung cancer, with adenocarcinoma (LUAD) and squamous cell carcinoma (LUSC) being its major subtypes. Smoking alone cannot completely explain the lung cancer etiology. We hypothesize that altered lung microbiome and chronic inflammatory insults in lung tissues contribute to carcinogenesis. Here we explore the microbiome composition of LUAD samples, compared to LUSC and normal samples. Extraction of microbiome DNA in formalin-fixed, paraffin-embedded (FFPE) lung tumor and normal adjacent tissues was meticulously performed. The 16S rRNA product from extracted microbiota was subjected to microbiome amplicon sequencing. To assess the contribution of the host genome, *CD36* expression levels were analyzed then integrated with altered NSCLC subtype-specific microbe sequence data. Surprisingly phylum *Cyanobacteria* was consistently observed in LUAD samples. Across the NSCLC subtypes, differential abundance across four phyla (*Proteobacteria, Bacteroidetes, Actinobacteria*, and *Firmicutes*) was identified based on the univariate analysis (*p*-value < 6.4e-4 to 3.2e-2). *In silico* metagenomic and pathway analyses show that presence of microcystin correlates with reduced CD36 and increased PARP1 levels. This was confirmed in microcystin challenged NSCLC (A427) cell lines and *Cyanobacteria* positive LUAD tissues. Controlling the influx of *Cyanobacteria-like* particles or microcystin and the inhibition of PARP1 can provide a potential targeted therapy and prevention of inflammation-associated lung carcinogenesis.

## Introduction

The leading cause of cancer deaths worldwide is lung cancer with an annual death rate of 1.59 million (Stewart, 2014). Non-small cell lung cancer (NSCLC) constitutes more than 80% of the disease, with adenocarcinoma (LUAD) and squamous cell carcinoma (LUSC) being the major forms of NSCLC. The leading cause of lung cancer is cigarette smoking (Dela Cruz et al., 2011; Hecht, 2012), but other carcinogens and hereditary factors significantly contribute to occurrences. Five-year survival for NSCLC is <50% post-surgical resection and other interventions like chemotherapy (Giaccone, 2002). To better diagnose and manage individualized therapy for NSCLC patients, identification of reliable biomarkers that can untangle the complex heterogeneity of lung cancer subtypes is necessary (Yanagisawa et al., 2007).

Microbial pathogens have been linked to respiratory diseases. Identification of these pathogens in lungs was based on bacterial culture and staining techniques that could reliably identify only small numbers of microorganisms. Since over 70% of human microbial species are not culturable by standard microbiology media, errors and omissions in disease-microbe associations that can impact patient treatment are highly likely (Suau et al., 1999; Dickson et al., 2013). High-throughput sequencing has identified associations between specific human microbial species and diseases like obesity, diabetes, atherosclerosis, colorectal cancer, cystic fibrosis and colitis (Turnbaugh et al., 2006, 2009; Castellarin et al., 2012; Karlsson et al., 2012, 2013; Lu et al., 2016).

Saliva, sputum and bronchoalveolar lavage have been used to detect disease-related microbes (Hoebe et al., 2005; Koch et al., 2011; Karlsson et al., 2013), and reports link inflammatory pathways to cancer (Kundu and Surh, 2008; Nathan and Ding, 2010). An important mediator of these inflammatory pathways is the scavenger receptor *CD36*, which exerts anti-angiogenic responses and promotes pro-inflammatory signals that can lead to chronic inflammation (Koch et al., 2011). CD36 signaling has been shown to be initiated by pathogen-derived ligands or toxins from *P. falciparum, M. pneumoniae*, and *S. aureus* (McGilvray et al., 2000; Hoebe et al., 2005; Stuart et al., 2005). *CD36* receptors [(which also behave like toll-like receptors (TLRs)] have been implicated in both innate and adaptive immune responses through their recognition of pathogens and pathogen-associated molecules, including Gram-negative lipopolysaccharide of *Neisseria meningitides* and Gram-positive *Staphylococcus aureus* and *Listeria monocytogenes* (Zhong et al., 2013). In lung tissues, altered expression of *CD36* is associated with lung cancer (Nakamura et al., 2003; Mehan et al., 2012). CD36 exerts anti-angiogenic responses and inflammatory roles (Koch et al., 2011). We hypothesize that CD36 could provide a connection between lung microbiota and particulate insults that contribute to lung cancer development.

There are very sparse reports to date that have identified microbes that reside inside or infect lung cancer cells *per se*. Currently, genes that are used as biomarkers are inadequate in explaining the development of the different lung cancer types (Weiss et al., 2010; Sequist et al., 2011; Bergethon et al., 2012). This insufficient knowledge of the factors that may explain the etiology of lung cancer types (Pesch et al., 2012), limits the ability of clinicians to detect and classify NSCLC subtypes, like LUAD and LUSC (Dickson et al., 2013; Schwabe and Jobin, 2013). This further halt effective prevention and treatment of the diseases. Recent studies have shown that stress due to environmental factors can alter patients lung microbiome (Pauly and Paszkiewicz, 2011; Garmendia et al., 2012; Dickson et al., 2013; Fulbright et al., 2017). Response to these environmental influences can disparately vary by population and/or geographical region (Busch et al., 2016). Major gaps still exist in understanding the myriad roles of the microbiome (Bhatt et al., 2017; Fulbright et al., 2017) during lung cancer progression. Particularly in patients of different geographical regions and climate. Recently, the microbiome of non-malignant lung tissue samples has been characterized (Yu et al., 2016). Microbial composition and tissue-specific microenvironments can significantly affect the behavior of primary pathogen and disease conditions (Duan et al., 2003). Therefore, analysis of microbial profiles inside the lung cancer cells may have significant clinical implications.

We used a cross-sectional study design and retrospectively collected formalin-fixed, paraffin-embedded (FFPE) tissues that were confirmed NSCLC clinical phenotypes. For each patient tumor tissue and adjacent normal tissues were used and subjected to 16S rRNA sequencing to provide a snapshot of the microbiota composition in LUAD and LUSC compared to the adjacent normal tissues. We used the microbiome profiles to predict microbial enrichments associated with the NSCLC subtypes.

## Materials and methods

To assess the composition of microbiota in the NSCLC tissues, a cross-sectional (or a snapshot) approach was used to evaluate the change in microbiota profile as exposures contributing to the development of LUAD and LUSC.

### Sample acquisition

Lung biopsies are better than other sample types in terms of avoiding oral contamination. In addition, low biomass lung sample types, such as BAL fluid containing low environmental contamination may contribute dominantly to the PCR or Next Generation Sequence (NGS) results. Therefore, lung biopsy samples of patients undergoing surgery were collected by experienced surgeons and placed in a sterile optimal cutting medium (OCT) then flash frozen in liquid nitrogen. Flash freezing in liquid nitrogen maintains the integrity of the sample for downstream analysis, e.g., PCR, microbiology, and biochemistry. Samples in the OCT are then stored at −80 for the long term. Portions of the samples that are embedded in OCT are sliced for histological analysis and for making formalin fixed paraffin embedded (FFPE) blocks using new nuclease-free reagents. These FFPE lung tissue samples were 1–5 years old and obtained from 50 to 80 years patients of African and European ancestry. All samples were collected with informed consent approved by the University of Arkansas for Medical Sciences (UAMS) Institutional Review Board. Tissue blocks were sectioned at 2 μm and were stained with hematoxylin and eosin (H&E) to identify the tumor and the normal area in each block. Histopathologic evaluation was performed to assess the percentage of tumor in each tissue samples from both the tumor and normal tissue compartments. Sections of 5 μm were used for DNA, RNA, protein microbiome analyses. We used 11 LUAD, 10 LUSC, and 8 adjacent normal FFPE samples, derived from patients treated at the UAMS to survey for microbial profiles. All this work was done under sterile gloves, scalpel, and forceps. All that said, the wet-lab practice is usually challenging to obtain absolutely contaminant-free DNA/RNA from samples. Therefore, to further filter signal from possible contamination from reagents and equipment, we used blank reagents as controls in our analysis.

### Extraction of total (genomic and microbial) DNA, RNA, proteins from FFPE and fresh frozen NSCLC samples

We acknowledge the fact that bacterial contamination can occur due to sample collection and handling at every step of the pipeline which can lead to sources of variation in microbiota profiling. To avoid contamination from other exogenous nucleic acids: DNA and RNA, microbial nuclease-free water that has been filter-sterilized and UV-treated was used. The quality of the water was then tested by PCR using microbial DNA primers before use. Further, work surfaces were decontaminated by washing with 10% chlorine to hydrolyze possible DNA contaminants. Nucleic acid free reagents and aerosol resistant pipette tips were used. All sample racks and reusable equipment were also washed in 10% chlorine and autoclaved after use. 10% chlorine was used to spray pipettors and working areas then placed in the UV chamber for at least 30 min after and before use to destroy DNA. Mixing and aliquoting pre-amplification ingredients were done on the bench top of the UV cabinet. In addition, we derived our negative controls from biopsied paraffin block adjacent to the lung tissues and from sterilized and microbial nuclease-free filtered water used in our reagents. For the FFPE samples, total DNA was isolated from samples using AllPrep DNA/RNA FFPE kit (Qiagen) and QIAamp DNA microbiome kit (Qiagen) following manufacturer's protocol with modifications. For the fresh frozen samples, DNA, RNA and protein were extracted using Qiagen's AllPrep DNA/RNA/Protein Mini Kit (Orloff et al., 2012) (Qiagen, Valencia, CA, USA) according to manufacturer's protocol. The extracted DNA was quantified using the NanoDrop Lite spectrophotometer (Thermo Scientific), which ranged from 30 to 98 ng/μl. The availability of microbial DNA was verified by PCR amplification using 16S rRNA specific primers, 27F (5′-AGAGTTTGATCMTGGCTCAG-3′) and 519R (5′-GWA TTA CCG CGG CKG CTG-3′). As a quality check, we used commercial genomic DNA as our negative control, microbial DNA derived from the FFPE samples, host human genomic DNA from the same FFPE samples that served as the second negative control, and nucleic acid-free filtered water (blank) as a negative control. We expected to see bacterial 16S rRNA 500 base pair (bp) amplicon amplifying in the microbial DNA derived from the FFPE samples. All the other samples: host genomic DNA, commercial genomic DNA, and Nuclease free water were expected to be negative for the 500 bp amplicon. Samples that were positive for bacterial 16S rRNA 500 bp amplicon (Figure S1) were then subjected to 16S rRNA sequencing.

### 16S rRNA sequencing

FFPE samples derived from the 11 LUAD, 10 LUSC, and 8 adjacent normal tissues were then subjected to a two-step process following the Illumina protocol. Exactly 500 bp of 16S rRNA genes were amplified with the universal eubacterial primers 27F (5′-AGAGTTTGATCMTGGCTCAG-3′) and 519R (5′-GWA TTA CCG CGG CKG CTG-3′) (Petti et al., 2005) using the high-fidelity AB-gene DNA polymerase (Thermo Scientific) using [95°C for 3 min, 30 cycles (95°C for 30 s, 55°C for 30 s,72°C for 30 s), 72°C for 5 min then finally Hold at 4°C] as the PCR condition. A secondary amplification was then done using [95°C for 3 min, 8 cycles (95°C for 30 s, 55°C for 30 s, 72°C for 30 s), 72°C for 5 min then finally Hold at 4°C] was performed in where the universal primers were modified to contain the Illumina sequencing adaptors A and B and an 8-bp “barcode” specific to each sample (McKenna et al., 2008). These were then sequenced on the Illumina MiSeq with 150 bp paired-end reads. All experimental procedures in this section were done at the UAMS Sequencing Core Facility that routinely does next-generation sequencing services and has an established SOP.

### Classification of microbial 16S sequences into operational taxonomic units

Sequences were demultiplexed and aligned to 16S rRNA sequence database (Greengenes version May 2013) (DeSantis et al., 2006) and clustered into Operational Taxonomic Units (OTU) at 97% sequence identity using QIIME bioinformatics pipeline version 1.9.1 (Caporaso et al., 2010). We observed an average 540,159 sequences per sample (see Table S2 for the number of sequences per sample). We followed a closed reference OTU picking strategy. To account for biases caused by uneven sequencing depth, equal numbers of random sequences (=238,320) were selected from each sample prior to calculating community-wide dissimilarity measures. One sample that produced only 32,846 sequences was removed.

The OTU table in a BIOM format (Biological Observation Matrix 1.0.0) was imported to an R suite environment through PhyloSeq package (McMurdie and Holmes, 2013) for statistical analysis and illustration of results. To identified the key OTUs associated with a different group, we used the PLS-DA analysis (Barker and Rayens, 2003) and calculated VIP scores (Wold and Cocchi, 1993) using ropls R version 3.4.2 and, PhyloSeq version 1.22.0 (Thévenot et al., 2015).

### Sanger sequencing to confirm the presence of a bacterial phylum

To confirm the presence of *Cyanobacteria* sequence in NSCLC tissue, we used previously reported *Cyanobacteria*-specific primers: CYA106F: CGG ACG GGT GAG TAA CGC GTG A and CYA781R (a): GAC TAC TGG GGT ATC TAA TCC CAT T to amplify a 750-bp amplicon (Benson et al., 1997; Maidak et al., 1997; Nübel et al., 1997). The resulting amplicons were run on a 1% agarose gel, purified using MinElute Gel Extraction Kit (Qiagen), subjected to standard directional Sanger sequencing using BigDye vs 1.1 (Applied Biosystems) and electrophoresed on a 3130XL Genetic Analyzer (Applied Biosystems). Sequence results were analyzed using Mutation Surveyor software and compared with reads from 16S rRNA sequencing and publicly available microbiome data at the Human Microbiome Project (http://hmpdacc.org/resources/data_browser.php).

### Analysis of the role of cyanobacteria toxins in lung cancer

The detection of *Cyanobacteria* in the LUAD samples led to the hypothesis of the possible existence of *Cyanobacteria* toxin as has been reported previously (Zanchett and Oliveira-Filho, 2013). Therefore, to find the link between *Cyanobacteria* toxin (i.e., mycrocystin) and the development of lung cancer we used bioinformatics software prediction tools and curated pathway databases such as MetaCore™ and Comparative Toxicogenomics Database. MetaCore harbors a sophisticated integrated pathway and network analysis for multi-omics types of data. The MetaCore platform provides a comprehensive systems biology analysis suite that aid identification of high quality experimental molecular interactions and pathways, gene disease associations, chemical metabolism and toxicity information. Analysis of the biological pathways, disease and gene network processes that are associated with microcystin were done.

### *In silico* reconstruction of metagenomic pathways to define the general bacterial community function in the lung cancer microenvironment

Since not any one bioinformatics tool or database can provide comprehensive information, we used multiple data sources like MetaCore and Comparative Toxicogenomics Database that complements or supplements each other and also enables us to get a consensus opinion on the key functional roles. Along these same lines, PICRUSt (phylogenetic investigation of communities by reconstruction of unobserved states) was used to predict the functional composition of metagenomes for each sample (Langille et al., 2013). This approach uses evolutionary modeling to predict metagenomes from 16S data and a reference genome database, useful for detecting microbial functions and their variability when the quantity of bacterial DNA present is low (Davenport et al., 2014). With PICRUSt, we were able to accurately map (generated 95% confidence intervals for each gene prediction) 16S microbial sequence reads to gene family abundances, which were then used to reconstruct pathways or functions using the metagenomics pathway tools. This helped acquire the general bacterial community function within the lung cancer microenvironment. The gene content estimations were mapped to KEGG pathways (Kanehisa et al., 2012) to identify functional enrichment of these pathways, and the Wilcoxon Rank Sum test was used to identify significant differences.

### Screening for the presence of microcystin in cyanobacteria positive LUAD samples

Extracted proteins from fresh frozen LUAD samples that harbored *Cyanobacteria* and also had matching FFPE samples were collected for analysis of microcystin content by ELISA. Additional and independent of LUAD samples, proteins from LUSC samples and negative controls were similarly targeted for analysis of microcystin. The QuantiPlate Kit for Microcystins High Sensitivity from EnviroLogix Inc. (Portland, ME) was used to perform the ELISA. Each sample was prepared from the protein extract, at a dilution factor of 1:25. Microcystin-YR (Sigma-Aldritch, Inc.) was used as a sample positive control, at concentrations of 1 ppb and 0.5 ppb to fall within the range of the microcystin ELISA kit. All dilutions were prepared using 10% (v/v) methanol in DI water. The ELISA was performed according to manufacturer's instructions (EnviroLogix Inc., LLC, Portland, ME). The ELISA assay was read using a SpectraMax M5 microplate reader (Molecular Devices, LLC, San Jose, California) at a wavelength of 450 nm. The results were analyzed using SofMaxPro software v. 6.5.1 (Molecular Devices, LLC, San Jose, California) provided with the microplate reader, using calibration controls as standards.

### Analysis of lung-associated CD36-specific expression

Previous genome-wide research showed that CD36 under express in lung cancer (Orloff et al., 2012). We decided to first confirm this finding using publicly available data prior to analyzing our precious samples. Therefore, *CD36* mRNA expression in lung cancer patients was extracted from publicly available GEO Dataset GSE1918 (Hou et al., 2010), which utilized the Affymetrix platform. There were 65 normal, 45 LUAD and 27 LUSC samples. *CD36* probe-specific levels were imported into R and quantile normalized (versions: R 3.2.3, Biobase 2.30.0, GEOquery 2.36.0, limma 3.26.8). Each probe was then analyzed for differential expression using empirical Bayes framework as implemented in R package *limma* (Ritchie et al., 2015) and false discovery rate adjusted *p*-values were reported. Normal lung tissues were compared to LUAD and LUSC tissues.

Then, similarly, we extracted mRNA from 71 fresh frozen lung tissues (i.e., 28 normal, 25 LUAD, and 18 LUSC), which included samples from 6 LUAD, 2 LUSC and 7 normal tissue matching compartments that were also simultaneously subjected to 16S rRNA sequencing. Using fresh frozen samples allowed extracting good quality and longer mRNA transcript that we were unable to get from FFPE samples. qRT- PCR reactions were performed on a 7900HT Fast Real-Time PCR System (Janabi et al., 2000) using FAST SYBR Green Master Mix (ABI) and developed primers (*HPRT1*: FWD-5′-TGGACAGGACTGAACGTCTT-3′, REV-5′-GGGCTACAATGTGATGGCCT-3′; *CD36*: FWD-5′-GCCATCTTCGAACCTTCACTAT-3′, REV-5′-GGTCTTCTAATGCAGTCGATTCT-3′). Experiments were performed in duplicates of the fresh frozen tissue samples. *HPRT1* was used as the housekeeping gene as it has been identified as being stable for use with lung samples (Liu et al., 2005). *CD36* expressions level fold changes were calculated relative to this gene using 2-ΔΔCT method (Livak and Schmittgen, 2001).

### To evaluate the role of microcystin in A427 cells and compared with *Cyanobacteria*-positive LUAD samples

A427 lung tumor cell lines were purchased from ATCC and cultured in standard incubation conditions using DMEM without glutamine (Sigma St. Louis, MO), 10% fetal bovine serum (FBS), and 1% penicillin-streptomycin (complete medium) at a humidified 37°C with 5% CO_2_. The cell line was propagated from an initial concentration of 100,000 cells per flask. Sub-cultures of the cell line were then seeded into 6 well cell culture plates at 100,000 cells/well. Cells were allowed to attach for 24 h. Media were removed from the flasks and the cell line was given 5 mL glutamine-free complete medium (DMEM without glutamine, 10% fetal bovine serum (FBS), and 1% penicillin-streptomycin) (Sigma St. Louis, MO). When the cells were at about 80% confluence, media was removed, and replaced with medium containing microcystin at different concentrations then incubated for 48 h. After treatment, cells were washed with ice-cold PBS, lysed with RIPA buffer then subjected to Western blot analysis. Western blot analysis was performed according to the methods described previously (Qian et al., 1998). Briefly, the cell lysates were resolved in 10% SDS-PAGE gel and then transferred to nitrocellulose membranes, followed by blotting with different antibodies for the individual targeted proteins. Horseradish peroxidase-conjugated secondary antibodies (Protein Simple) were applied to visualize proteins using chemiluminescence.

To further confirm if microcystin is embedded in selected LUAD sample, we selected LUAD samples that were positive for *Cyanobacteria*, and compared with LUSC samples, negative control and positive control (i.e., microcystin). The protein extracts from the samples were used to quantify microcystin. Similar to A427 cells proteins from the NSCLC samples were also subjected to Western blot analysis (Qian et al., 1998).

## Results

### Microbial population profiles in the NSCLC subtypes

**Figure 1:**
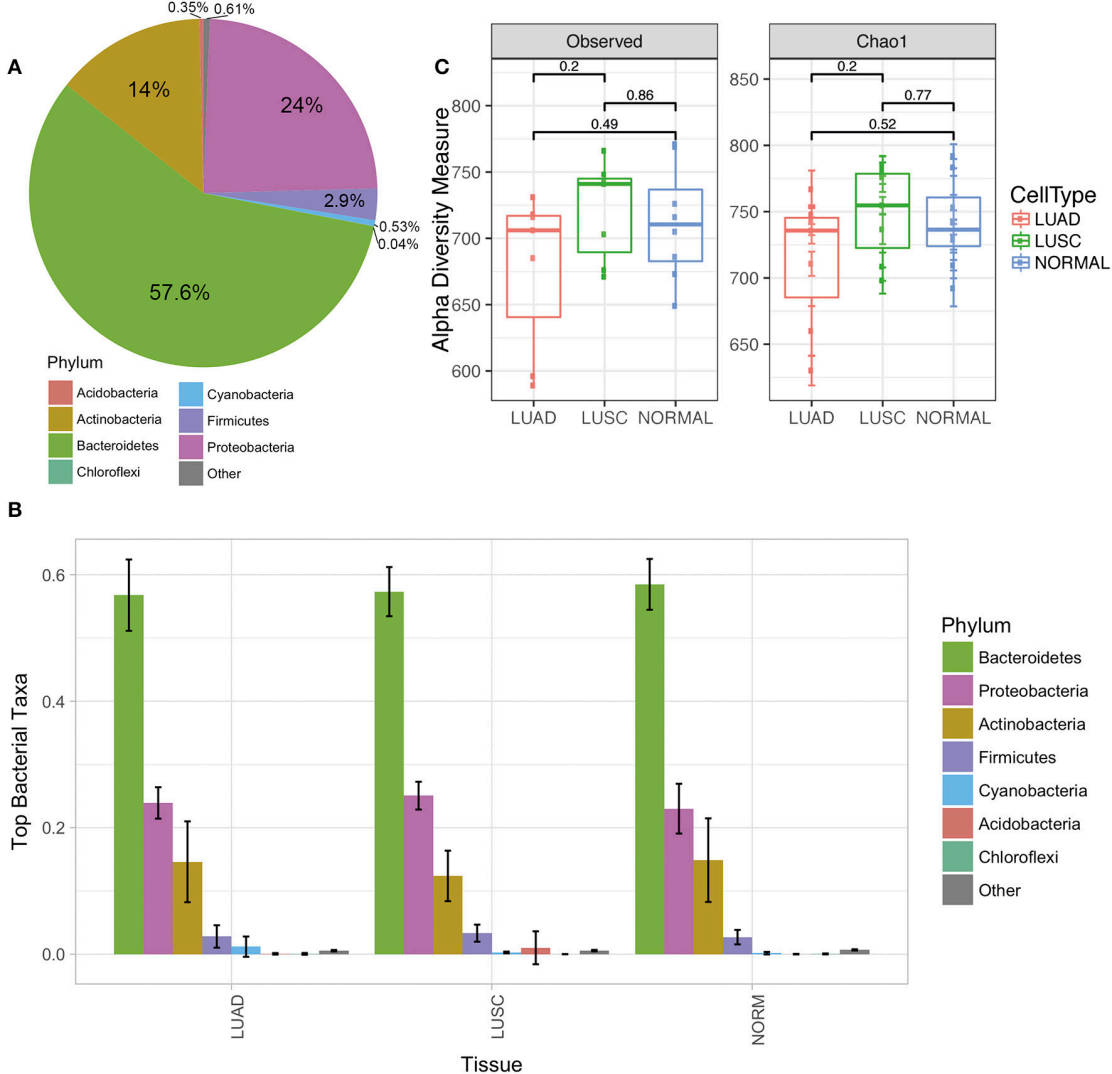
The diversity of lung microbiome derived from a normal cell and tumor cell of Non-small cell lung cancer (NSCLC) of the two subtypes adenocarcinoma (LUAD) and squamous cell carcinoma (LUSC). **(A)** The average of the phylum level pie chart of all lung samples in this study (*N* = 22). **(B)** Phylum level distribution (relative abundance) of microbial communities comparing paired normal (*N* = 8), LUAD (*N* = 7), and LUSC (*N* = 7) lung tissue patients (total *N* = 22). Each bar represents a fraction of the bacteria detected in a given sample. **(C)** Alpha diversity plot of the three groups of samples (normal, LUAD and LUSC) by observed and Chao1 model. The statistical comparisons of mean among the group were reported as *p*-values derived from Mann-Whitney test.

Analysis of microbiota from FFPE lung cancer samples revealed differences between normal and tumor samples. Seven phyla were identified via amplicon sequencing of 16S rRNA (Figure 1A). *Bacteriodetes* and *Proteobacteria* were the most predominant phyla in patient lung samples, accounting in average for 57.6 and 24% respectively. Other phyla identified included *Actinobacteria* 14%, *Firmicutes* 2.9%, *Cyanobacteria* 0.53%, *Acidobacteria* 0.35 and *Chloroflexi* 0.04% (Figure 1A).

After stratifying NSCLC by subtypes, we observed that the abundant phylum levels were similar (Figure 1B). However, we observed differences as we compared phyla through OTUs identified under each phylum (see below). The richness of individual sample was estimated through observed alpha diversity and Chao1 method (Figure 1C). There were no significant differences among the three groups of samples; however, the alpha diversity of LUAD was slightly lower than LUSC and normal samples.

**Figure 2:**
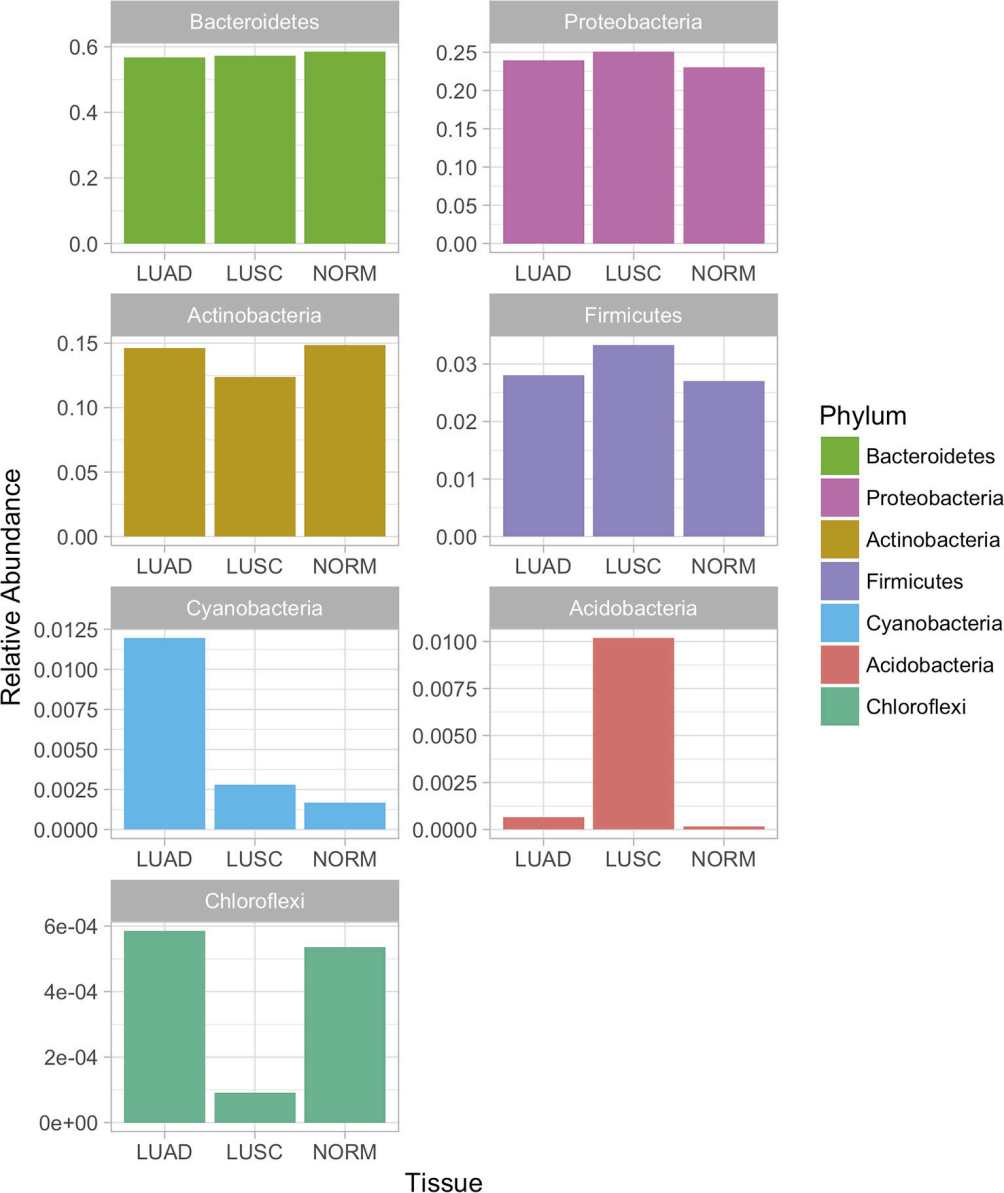
Relative abundance of bacterial phyla in the microbiota of lung cancer subtypes and normal lung samples. Differential abundance across phyla were identified based on the univariate analysis (Mann-Whitney test). *Actinobacteria* (LUAD vs. normal *p*-value < 3.2e-2, LUSC vs. normal *p*-value < 13.2e-4), *Bacteriodetes* (LUAD vs. normal *p*-value < 3.8e-5, LUSC vs. normal *p*-value < 3.6e-3) were significantly different when both subtypes of NSCLC were compared with normal. *Firmicutes* (*p*-value < 1.3e-5) and *Proteobacteria* (*p*-value < 6.4e-4) were significantly different in abundance then the NSCLC subtypes.

Considering the relative abundances of the phyla detected in our samples across three sample groups (Figure 2), some differential abundance across phyla were identified based on the univariate analysis (Mann-Whitney test). *Actinobacteria* (LUAD vs. normal *p*-value < 3.2e-2, LUSC vs. normal *p*-value < 13.2e-4), *Bacteriodetes* (LUAD vs. normal *p*-value < 3.8e-5, LUSC vs. normal *p*-value < 3.6e-3) were significantly different when both subtypes of NSCLC were compared with normal. Interestingly, *Firmicutes* (*p*-value < 1.3e-5) and *Proteobacteria* (*p*-value < 6.4e-4) were significantly different in abundance then the NSCLC subtypes. Both phyla showed low levels in LUAD compared to LUSC and normal samples. Altered levels of *Proteobacteria, Bacteroidetes, Actinobacteria*, and *Firmicutes* in the LUAD patient samples correlate with the abundance of phylum *Cyanobacteria* that are uniquely predominant in the LUAD samples (Figures 1B, 2).

**Figure 3:**
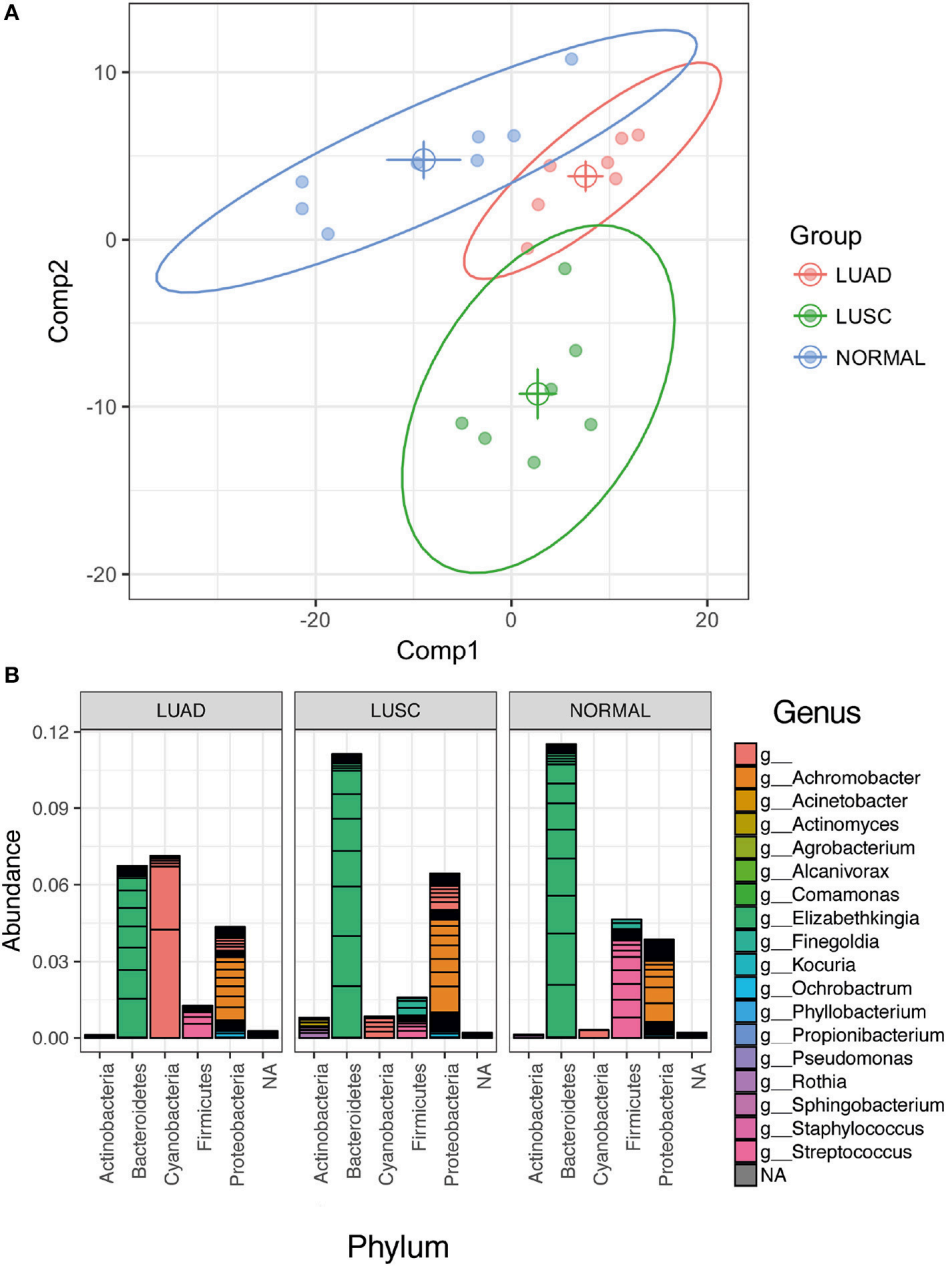
Multivariate analysis using PLS-DA (Partial Least Squares Discriminant Analysis) method. **(A)** PLS-DA loading plot shows a good discrimination of the three groups of samples (normal, LUAD and LUSC). The solid circles represent individual samples. The centroid of the individual group was calculated and plotted as open circles. The 95% confidential ellipse of individual group was plotted as a solid line. **(B)** Stack OTUs bar plot of the relative abundance of bacterial 1048 phyla based only the OTUs that have VIP values > 1.5.

Further, partial least square discrimination analysis (PLS-DA) (Schwabe and Jobin, 2013), which is a supervised method of multivariate analysis, was employed to identify the key OTU that contribute to discriminate the three groups of the sample types apart. As seen in Figure 3A, the loading plot obtained by PLS-DA model showed separation of samples into the LUAD, LUSC and normal distinct clusters. Variable Importance in Projection (VIP) scores (Pauly and Paszkiewicz, 2011) of the PLS-DA model were calculated to evaluate the importance of individual OTU on the discrimination. At the cut-off of VIP score > 1.5, which was considered to be a good feature, 42 OTUs were identified. The 42 OTUs are classified into 18 known genera of known 5 phyla of *Proteobacteria, Bacteroidetes, Actinobacteria, Firmicutes*, and *Cyanobacteria* (Figure 3B)*. Cyanobacteria* were identified within agreement by the univariate (*p*-value was derived from the statistical test) and multivariate (i.e., high VIP from PLS-DA) indicating the high confidence of our finding.

Many of the bacterial genera that we identified by the PLS-DA in the lung tissue of patients (Figure 3B) have also been reported in previous studies in patients with lung cancer and lung diseases. Our studies show a similar microbial composition, with *Achromobacter, Acinetobacter, Actinomyces, Elizabethkingia, Rothia, Sphingobacterium* in lung cancer patients (Wallet et al., 1997; Colmegna et al., 2003; Claassen et al., 2011; Lin et al., 2016). We also detected potential opportunistic pathogens such as *Kocuria, Pseudomonas, Staphylococcus, and Streptococcus* which were all reported in the presence of lung cancer (Berghmans et al., 2003; Ahmed et al., 2014). In addition, two genera, *Propionibacterium* and *Ochrobactrum*, appeared in both normal lung tissue and lung cancer patients (Ishige et al., 2005; Cameron et al., 2017). This suggests that our observations represent an accurate reflection of the bacterial composition of the lung microbiome. In addition to lung cancer, *Finegoldia, Phyllobacterium, and Sphingobacterium* have also been reported to be correlated with other lung diseases (Davis and Systrom, 1998; Lambiase et al., 2009; Boutin et al., 2015).

Additionally, the relative abundance of *Cyanobacteria* in LUAD was higher than LUSC and normal samples (Figure 3B). Moreover, the genus information of the identified *Cyanobacteria* lacked in details. The OTU consensus sequences from Phylum *Cyanobacteria* (see Supplementary Materials) were searched against Genbank database that contains more taxonomic collection than Greengenes Database that we used for standard OTU picking. The best hits of all consensus sequences are 16S rRNA of “Uncultured cyanobacterium” (GQ502588.1, FJ024312.1, KU667126.1 and KM892905.1) with identity >92% and *p*-value < 1.57e-178. All these sequences link to the *Taxonomy ID:* 1211 that shows the full lineage as (cellular organisms; Bacteria; Terrabacteria group; *Cyanobacteria*/ Melainabacteria group; *Cyanobacteria*; environmental samples). Based on this knowledge, we further investigated the existence of *Melainabacteria* in our samples which have been proposed to be closely related to *Cyanobacteria* with a common ancestry (Soo et al., 2014, 2017). The evolution of *Cyanobacteria* is still a mystery, as relatives to this phylum have not been well characterized. Recent studies have suggested that *Cyanobacteria* split to form a closely related phylum *Melainabacteria* prior to the acquisition of oxygenic photosynthesis properties. We have observed trace amounts of *Melainabacteria* only in LUSC but not in LUAD and Normal samples (Sequences classified against Silva rRNA Database version 132). LUAD samples had a higher level of *Cyanobacteria* (LUAD vs. LUSC *p*-value < 0.056; LUAD vs. NORMAL *p*-value < 0.039) and we did not see a difference between LUSC and Normal samples (*p*-value = 0.317). These *Cyanobacteria sequences are closely related to the Cyanobacteria*/ Melainabacteria group. This strongly supports our finding of the presence of *Cyanobacteria* in LUAD, thus the need for further investigation of *Cyanobacteria and/or Melainabacteria* and its toxins.

### Confirmation of the presence of cyanobacteria in LUAD through direct sequencing

To test our findings, we amplified sample 9374-S4-LUAD (which had ~5% *Cyanobacteria)* and sample 9378-S3-LUAD (which showed 0.0% *Cyanobacteria)* based on the MiSeq data. The 0.0% *Cyanobacteria* read was chosen so as to track small amounts in the LUAD patients which was not captured by the MiSeq high throughput approach. Sample 9374-S4-LUAD amplified at the correct size (700 bp) and sample 9378-S3-LUAD showed a much lower amplification amount proportional to levels observed via MiSeq (Figure S1). A blank negative control and total human genomic DNA demonstrated no amplification of *Cyanobacteria*. The PCR products were excised from the gel, purified, sequenced, and blasted against 16S rRNA gene sequence database, demonstrating the sequences were of 16S rRNA of *Cyanobacteria*, validating our data. Since the negative controls produced no PCR products, we could not perform gel extraction and sequencing on the negative controls. Given the sensitivity of the MiSeq technique and since any contamination in the laboratory and of reagents have been known to impact microbiome data, sterile procedures were meticulously adopted together with including negative control samples (as mentioned above), even though *Cyanobacteria* is not among the contaminating phylum (Salter et al., 2014). As shown in Figure S2, a 700 bp band was amplified in the tissue samples but was absent in the adjacent paraffin material without tissue (negative control) and sterile filtered water samples. When the negative controls were sequenced, they were also negative for microbial sequences.

### The role of microcystin in the lung tissues

The detection of *Cyanobacteria* sequences in the LUAD samples led to the hypothesis of the possible existence of *Cyanobacteria* toxin (i.e. microcystin) as has been reported previously (Zanchett and Oliveira-Filho, 2013). Microcystin are a family of polyketide/peptide-derived toxins and *Cyanobacteria* are a prominent source of such compounds (Zanchett and Oliveira-Filho, 2013). Along these lines, the link between microcystin and inflammatory processes was further assessed using bioinformatics software prediction tools and curated pathway databases within MetaCore™ and Comparative Toxicogenomics Database harbors a sophisticated integrated pathway and network analysis for multi-omics types of data that identify the top enriched pathways, processes, and diseases associated with microcystin genes and CD36 genes.

**Figure 4:**
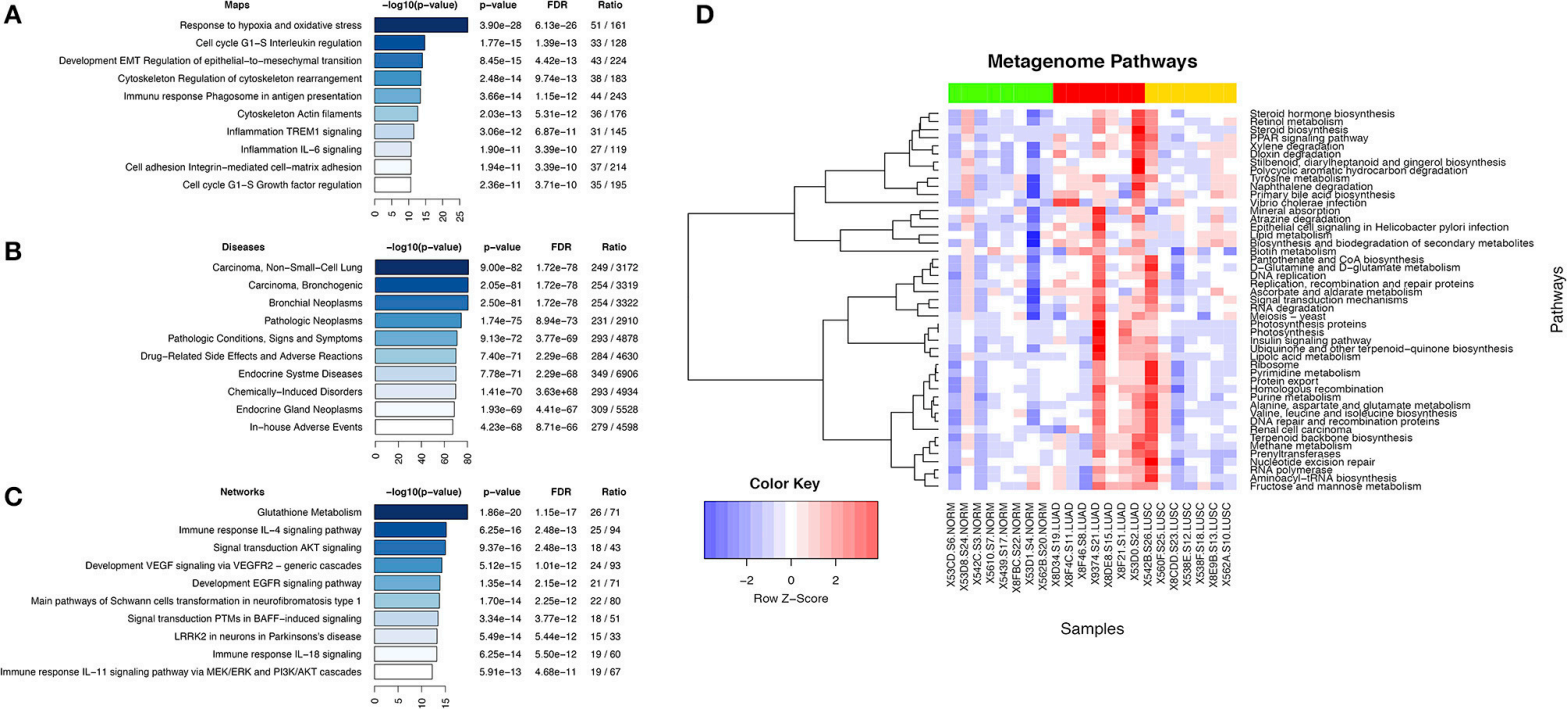
Display of microcystin related pathways in lung cancer. **(A)** Top ten enriched pathways maps of microcystin genes in *Cyanobacteria***; (B)** Enriched diseases for microcystin genes in *Cyanobacteria;* **(C)** Enriched process networks for microcystin genes in *Cyanobacteria*; **(D)** Metagenome pathways. The results of metagenome functional content prediction are shown. The metagenome content is annotated with KEGG pathways and comparison of the pathways in two groups are made (Wilcox rank sum test). The heatmap depicts only significant differences in pathway enrichment (*p*-value < 0.05). The heatmap colors show increased activity in pathways with red, and lower activity with blue. Normal samples (green color bar at the top) vs. LUAD samples (red color bar) vs. LUSC samples (yellow color bar) are shown.

Analysis of genes or gene products that interact with microcystin revealed top ten enriched pathways, functionally enriched process networks, enriched diseases, and enriched biological process networks (Figures 4A–C). Some of the pathways include Glutathione metabolic pathway, AKT signaling, EGFR signaling, response to hypoxia and oxidative stress, Cell cycle regulation, etc. are known to play a critical role in antioxidant defense, detoxification mechanisms, cell survival, proliferation, oncogenesis and NSCLC progression (Wagner and Schmidt, 2011; Guo et al., 2015). Further, the deregulation of many signaling pathways such as EGF/RAS/RAF/MEK/ERK and PI3K/AKT/mTOR is considered to play a critical role in oncogenesis and cancer progression (Memmott and Dennis, 2010).

### Predicted metagenomic pathways to define the general cyanobacterial community function in the lung cancer microenvironment

*Cyanobacteria*-specific sequence reads were sorted into significant metabolic/metagenomic pathways based on KEGG pathways and was compared between the lung cancer subtypes. We predicted the composition of the metagenomes first and mapped these to pathways. LUAD samples demonstrated significant differences in pathway assignments based on sequence reads when compared to other samples (Figure 4D). The top pathways of interest (Figures 4D) included PPAR signaling pathway (*p*-value < 0.0321), D-Glutamine and D-glutamate metabolism (*p*-value < 0.0177), Stilbenoid, diarylheptanoid and gingerol biosynthesis (*p*-value < 0.0092). The PPAR signaling pathway which has pro-inflammatory roles had increased activity in LUAD samples (Figure 4D). It is important to note that MetaCore, Comparative Toxicogenomics Database and Metagenomics pathway prediction in PICRUSt analyses gave overlapping pathways: the glutathione metabolism, signal transduction and cell cycle regulation.

### Presence of microcystin in cyanobacteria positive LUAD samples

**Figure 5:**
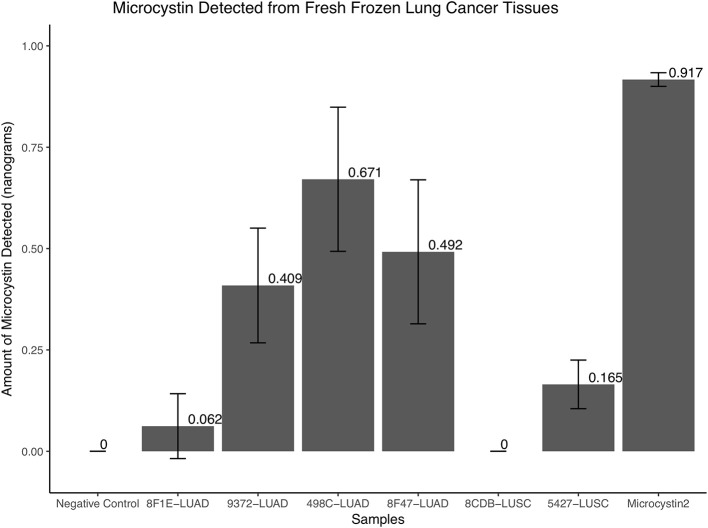
The quantity of microcystin present in fresh frozen lung cancer tissues. The two LUAD samples that were positive for *Cyanobacteria* in the FFPE samples showed a presence of microcystin (0.062 and 0.409 ng, respectively) and so were the other LUAD samples (i.e., 0.671 and 0.492 ng). The negative control and one of the LUSC (8CDB) sample did not show detectable amounts of microcystin, while we detected trace amounts of microcystin in LUSC sample 5427.

To further confirm if microcystin is embedded in selected two LUAD sequenced samples that were positive for *Cyanobacteria*, two randomly picked recent LUAD samples, two LUSC samples (one from the normal compartment and the other from the tumor compartment), a negative control and positive controls (microcystin) were used. The protein extracts from the samples were used to quantify microcystin as discussed in the Methods section. The two LUAD samples that were positive for *Cyanobacteria* showed a presence of microcystin (0.062 and 0.409 ng, respectively) and so were the other LUAD samples (i.e., 0.671 and 0.492 ng). The negative control and one of the LUSC (8CDB) sample did not show detectable amounts of microcystin, while we detected trace amounts of microcystin in LUSC sample 5427, Figure 5.

### CD36-expression in GSE19188 publicly acquired dataset and fresh frozen NSCLC tissues

As a follow-up from our previous study, we opted to validate the inflammatory role of CD36 Orloff et al. (2012) in a different series of NSCLC patient samples. Analysis of *CD36* expression patterns within the NSCLC tissues that harbor microbial insults can provide the link between patient inflammatory molecules and microbes in lung cancer patients. Therefore, to investigate *CD36* expression pattern in LUAD, we extracted probe-specific expression array data on the candidate gene *CD36* from publicly available GEO dataset, Hou et al. (GSE19188) (Hou et al., 2010). *CD36*-specific differential expression analysis comparing normal and LUAD samples (Table S1) demonstrated a significant decrease in *CD36* expression in LUAD tissues as compared to normal tissues for all probes utilized (adj-*p*-value < 2.91E-06). LUSC tissue also demonstrated a decrease in *CD36* as compared to normal tissues.

**Figure 6:**
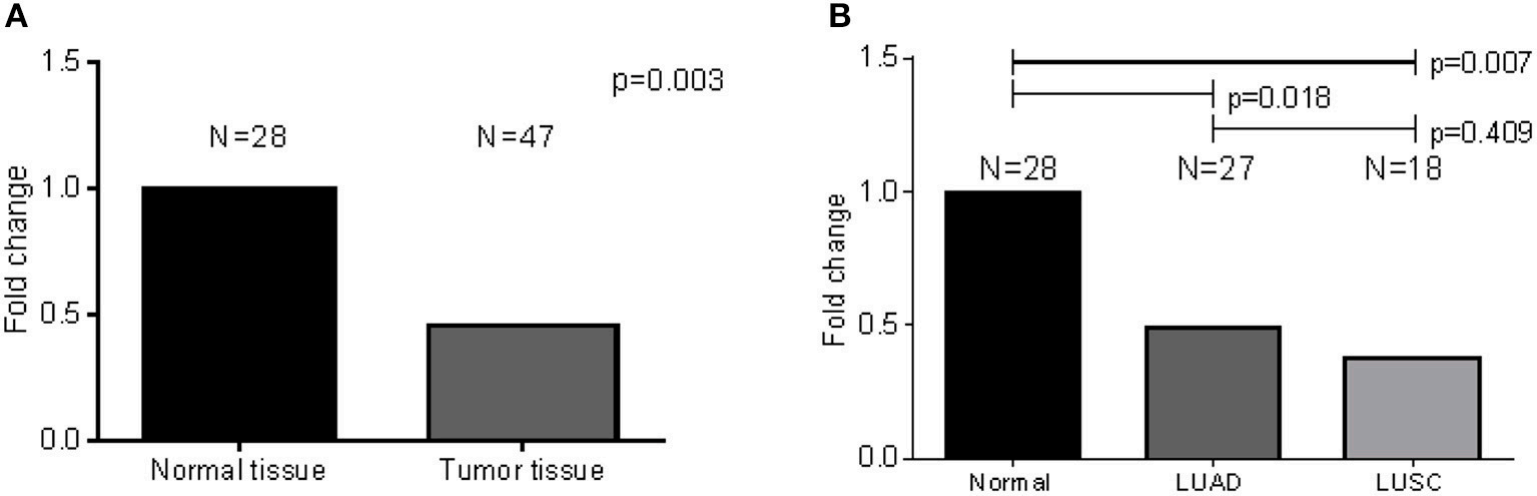
*CD36*-specific differential expression analysis comparing normal and LUAD fresh frozen samples. (A) Significant decrease in *CD36* expression was observed in LUAD tissues as compared to normal tissues. **(B)** There was no difference when LUAD was compared to LUSC.

To validate the findings from the publicly available data above, we analyzed *CD36*-specific mRNA levels derived from our fresh frozen lung tissue samples. A relatively less abundant mRNA is expressed in tumors when compared to normal tissue (results: *N* = 28; *p*-value < 0.003) (Figure 6). When a similar analysis was performed to compare normal tissue to tumor tissue by subtype, expression was significantly lower for LUAD (*N* = 27; *p*-value < 0.018) and LUSC (*N* = 18; *p-*value < 0.007) as compared to normal tissues, but not for LUAD as compared to LUSC (*p*-value = 0.409) (Figure 6). Note that 6 LUAD, 2 LUSC and 7 normal tissue compartments which were subjected to RNA expression analysis were also subjected to 16S rRNA sequencing, simultaneously.

### CD36, PARP1, and HNF4α levels in microcystin challenged NSCLC (A427) cell line and in LUAD cyanobacteria positive tissues

**Figure 7:**
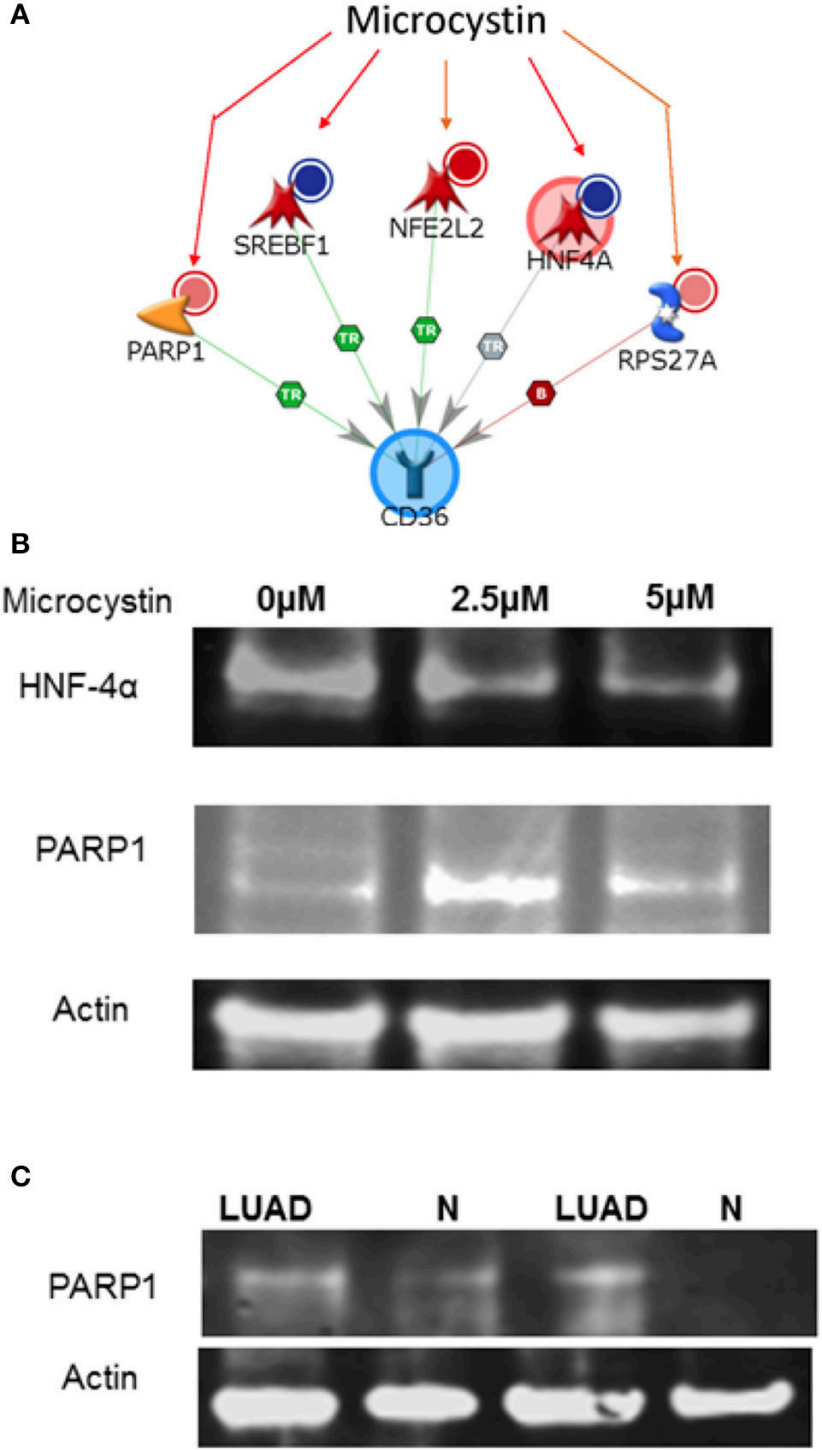
The interaction between CD36 and microcystin. **(A)** The exposure of microcystin, decrease expression of (i) SREBF1 mRNA, (ii) decrease expression of HNF4A mRNA; decreases degradation of (iii) of NFE2L2; **Increases** the expression of (iv) RPS27A; **increases** the cleavage of (v) PARP1 protein. **(B)** Microcystin differentially regulates PARP1 and HNF4α expression in A427 lung cancer cell line and in *Cyanobacteria* positive LUAD tissue samples. A427 cells were treated with various concentrations of Microcystin for 48 h, total cell lysate extracted and analyzed by immunoblotting using antibodies specific for PARP1 and HNF4α, (also see Figure S4). **(C)** Protein lysate was extracted from fresh frozen tissue-matched samples of patient positive for *Cyanobacteria* and immunoblotting analysis performed using a PARP1 antibody, (also see Figure S3).

There are about 600 genes known to be affected by Microcystin from studies on various organisms. These genes were uploaded onto MetaCore to identify top pathways, diseases, and processes that were affected. Upon exploring any direct interactions amongst Microcystin influenced genes and CD36 revealed 2 transcription factors (SREBF1 and HNF4A) and a ribosomal protein (RPS27A) that could possibly explain the suppressed expression of CD36 in patient samples. In addition, MetaCore predicted upregulation of Poly [ADP-ribose] polymerase 1 (PARP1) and down-regulation HNF4α proteins in the presence of microcystin toxin (Figure 7A). To verify and validate the MetaCore predictions, we challenged NSCLC cell line A427 with microcystin and performed western blot analysis using antibodies against PARP1 and HNF4α.

As shown in the results, microcystin exposure increased the PARP1 protein levels in the A427 cells and the level of HNF4α was reduced significantly, matching the MetaCore prediction, Figure 7B. Additionally, we tested the expression of PARP1 in LUAD tissue samples that were positive for *Cyanobacteria* and compared it to normal adjacent samples. PARP1 expression was higher in tumor samples compared to adjacent normal samples, Figure 7C. Taken together, this data shows that the *Cyanobacteria* toxin microcystin increases the development and progression of LUAD via PARP1 overexpression. Therefore, inhibition of PARP1 expression in combination with other therapeutic regimens should be considered in management and treatment of lung cancer and other forms of cancer (Wang et al., 2017).

## Discussion

The lung harbors an enormous internal surface area which is exposed to more than 8,000 liters of inhaled air daily that is carried through tiny alveoli (Dickson et al., 2014). The lungs are the human body's largest interface with the outside environment which harbors one of the most diverse microbiomes in the human body that includes viruses, fungi, bacteria, and toxins. Therefore, this interface is by no means a sterile surface (Dickson and Huffnagle, 2015). The lung microbiome of smokers vs. non-smokers has already been shown to be significantly different (Kim et al., 2017), but we further analyze the role of the NSCLC-specific microbiota (or its products) using 16S rRNA sequencing. The goal was to identify and analyze the microbiota that resides in the lung tissue that activates the inflammatory pathway leading to lung carcinogenesis. We found that the predominant phyla in the lung FFPE samples were *Actinobacteria, Proteobacteria*, and *Bacteriodetes*, constituting over 90% of all the phyla in our samples. The presence of *Cyanobacteria* sequences and/or microcystin in LUAD was also observed. The phylum *Cyanobacteria* was more abundant in LUAD compared to LUSC and normal samples. Analysis of disease and gene network processes that interacts with microcystin revealed glutathione metabolic pathway, AKT signaling, EGFR signaling, response to hypoxia and oxidative stress, cell cycle regulation, that play a critical role in detoxification mechanisms, proliferation, oncogenesis, and NSCLC progression. *In silico* microbial metagenomic analysis gave pathways that predicted the general *Cyanobacterial* community function in the lung cancer microenvironment revealed PPAR signaling pathway which has pro-inflammatory roles in LUAD samples. Presence of *Cyanobacteria* and microcystin likely, influenced the differential regulation of inflammatory molecules in the lung tissues: reduced the levels of CD36 (a toll-like receptor molecule), and increased PPAR1 levels in microcystin challenged NSCLC (A427) cell line and LUAD *Cyanobacteria* positive tissues. CD36 internalizes and processes *Cyanobacteria* microcystin residues in the lung alveoli, increasing PARP1expression that is important in cell proliferation and carcinogenesis (Choi et al., 2017; Luo et al., 2017).

**Figure 8:**
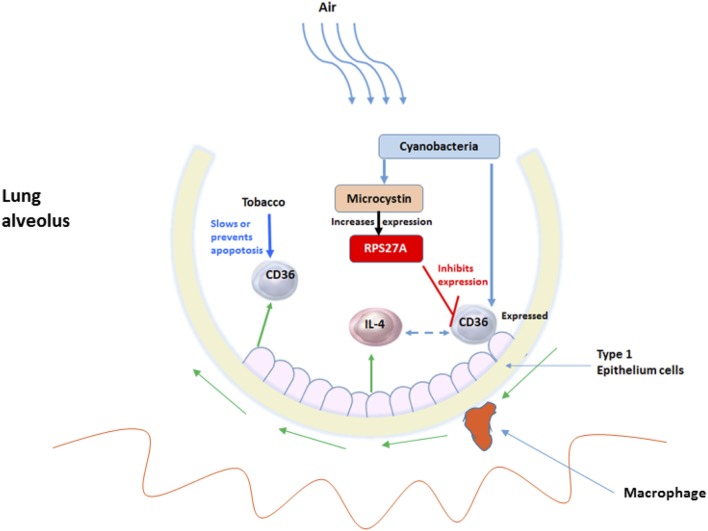
The interplay between *Cyanobacteria*, microcystin and inflammatory-related molecules in lung alveolus. The exposure of the environmental insults such as tobacco and microbes, for example, *Cyanobacteria* and its toxic microcystin and how they initiate the innate-inflammatory collaborative interaction to battle the insults. More specifically shows the involvement of the host-CD36 and cytokines and TLRs.

Lung cancer is an important cause of mortality worldwide. Of the 90% of lung cancer cases that are attributed to smoking, only 10–15% of smokers develop cancer, suggesting other influences, such as chronic lung inflammation (Houghton, 2013; Pevsner-Fischer et al., 2016). A recent study showed that an atmospheric concentration (mg/m^3^) of particulate matter of 10 mm in diameter (PM10) signifying considerable geographic variation can affect the lung microbiota (Yu et al., 2016). Further, PM2.5 seemed to play a crucial role in explaining the change in the composition of the lung microbiome (Ni et al., 2015). This data collectively suggest that geography and environment can alter the microbiome, which in turn can affect human health. The change in particulate matter in the environment within Arkansas warrants a deeper look into the related change in the microbial ecosystem and behavior. These changes may alter the quality of water and air that may affect the health of Arkansans. Along these lines, it is likely that microbes such as *Cyanobacteria* can be inhaled and stays in the lungs. In our study we detected *Cyanobacteria* sequences (Figures 1–3) and traces of microcystin (Figure 5) in the NSCLC tissues. Other reports have shown that people are most frequently exposed to harmful *Cyanobacteria* via contaminated water, orally, dermally and most importantly via aspiration to aquatic microbial communities containing *Cyanobacterial* cells and mixtures of cyanotoxins in untreated surface waters (Turner et al., 1990; Rapala et al., 2005; Stewart et al., 2006; Giannuzzi et al., 2011; Hilborn et al., 2014). Occasionally, these exposures have resulted in severe respiratory impairment characterized by pneumonia and adult respiratory distress syndrome (Turner et al., 1990; Giannuzzi et al., 2011). Hence, it is likely that the exposure or presence of *Cyanobacteria* and microcystin may influence inflammatory responses as we have attempted to show in Figures 7, 8.

Importantly, patients in this study identified as being smokers or users of tobacco originated from Arkansas. Arkansas is covered with large surface waters. There are six main rivers: Arkansas River, Mississippi River, Ouachita River, Red River, St. Francis River, and White River, which collect water from over 1,600 smaller watersheds throughout the state via tributary streams. The Arkansas River Valley, Gulf Coastal, and Delta eco-regions provide water that is conducive for agriculture a common trade in Arkansas Arkansas Department of Environmental Quality (2016). Along these lines, we expect to see increased use of pesticides and other intact agrochemicals, which have previously been identified in tobacco smoke (Borgerding and Klus, 2005) and have been linked to chronic inflammation in the lung. The lack of hygienic processes in agricultural practices can lead to different forms of contaminants, including microbiomes, which find their way into tobacco products. These contaminant microbes based on the Arkansas vegetation may include *Cyanobacteria*-like (i.e., Melainabacteria) (Soo et al., 2014, 2017) or microcystin that are inhaled into the lungs. Inflammation in the lung alveoli is mediated by different leukocyte subsets and different secreted factors. This continuous inflammation not only establishes a microenvironment that nurtures malignant transformation and tumor growth (de Visser and Coussens, 2006; Kundu and Surh, 2008; Nathan and Ding, 2010; Takeuchi and Akira, 2010) but also promotes specific microbial proliferation, Figures 7, 8.

The existence of *Cyanobacteria* in human body sites and more specifically in or around the lung tissues have been shown in earlier studies (Eckburg et al., 2005; Kim et al., 2009; Frank et al., 2010; Grice and Segre, 2011). In these studies, the human microbiota is primarily colonized by 6 phyla: *Firmicutes, Bacteroidetes, Proteobacteria, Actinobacteria, Fusobacteria*, and *Cyanobacteria. Cyanobacteria* have been shown to exist in nares (Kim et al., 2009). Most importantly, the lung microbiota is shown to have a higher relative abundance of *Proteobacteria, Thermi*, and *Cyanobacteria* (Yu et al., 2016). Given these earlier studies, we are more confident that our identification of *Cyanobacteria* sequences. The evolution of *Cyanobacteria* is still a mystery, as relatives to this phylum have not been well characterized. Recent studies have suggested that *Cyanobacteria* split to from a similar phylum *Melainabacteria* prior to the acquisition of oxygenic photosynthesis properties (Soo et al., 2014, 2017). *Melainabacteria* closely related to *Cyanobacteria* with a high sequence homology at specific sequence domains, but different form *Cyanobacteria* due to lack of photosynthetic capabilities. *Melainabacteria* are distinct from *Cyanobacteria* by containing flagella and lack of photosynthetic genes. *Melainabacteria* rely on anaerobic fermentation to generate energy. Not detecting traceable amounts of the *Melainabacteria* in LUAD samples does not mean it is absent in our samples this could be due the fact that we sequenced FFPE samples. Additional homology studies showed the *Cyanobacteria* sequences identified in LUAD had 92% homology to “uncultured cyanobacterium” (GQ502588.1, FJ024312.1, KU667126.1, and KM892905.1) that are in the *Cyanobacteria/Melainabacteria* group, Taxonomy ID: 1211. This strongly supports our finding of the presence of *Cyanobacteria* sequences in LUAD, thus the need for further investigation of *Cyanobacteria* and/or *Melainabacteria* and its toxins to help identify the disease processes we describe in this manuscript.

*Cyanobacterial* toxins either are membrane-bound or occur free within the cells. Studies have shown that most of the microcystin release occurs as bacterial cells age and die and passively leak their cellular contents (Sivonen et al., 1990). Further, microcystin can cross cell membranes into tissues through multi specific organic ion transport system (Runnegar et al., 1991) and where it can bind a 40 kilodalton protein a protein phosphatase 2A (PP2A) and possibly protein phosphatase 1 in the cytosol (Robinson et al., 1991). A similar ion transport system has been demonstrated in COPD mouse models exposed to smoking (Wallace et al., 2012). It is possible that we may have contaminants and we could also be dealing with non-photosynthetic Cyanobacterium. Even though Cyanobacterium is photosynthetic, it is highly likely that microcystin can find their way into the lung tissues. First, *Cyanobacteria* have been shown to be more toxic in warm climates, areas of good light intensity, and peaks during summer rather than winter (van der Westhuizen and Eloff, 1985). Therefore, conducive environmental conditions favor the proliferation of *Cyanobacteria* making it even easier for it or its product to be inhaled into the lungs. Identification of microcystin in the lung tissues maybe as a result of poor air and water quality in the region. *Cyanobacteria* cells can also be inhaled directly from untreated surface waters and the environment in these regions and some of the cells may remain alive while others die. The *Cyanobacteria* cells that remain alive and other microbiota create an ecosystem favoring competitive survival that leads to the cells producing microcystin that either stick onto the membrane or is channeled into the cytosol through active transport. This explains why we were able to detect microcystin in the LUAD tissues and probably specific features of LUAD favors *Cyanobacteria* accumulation or its toxin, Figure 5. Along these same lines, dead *Cyanobacteria* cells have been shown in previous studies to leak cyanotoxins hence this would be another way the microcystin can find its way into the lungs. In the surface waters, often *Cyanobacteria*'s inability to control its buoyancy may lead to its death (Ressom, 1994). Similarly, this lack of control of *Cyanobacteria* buoyancy may lead to its death in the lung tissues leading to the release of microcystin that either sticks onto the lung membrane or will be transported into the cytosol. Therefore, we believe there could be multiple sources of one getting infected with *Cyanobacteria* as stated above. Also, different forms of *Cyanobacteria* (i.e., dead or alive) can be inhaled and the cyanotoxin, microcystin, can stick on the membrane and/or transported into the cytosol of the lung cells. As stated above this was a snapshot or a hypothesis-generating study that warrants usage of a follow-up or observational study design that may validate our findings and decipher cancer-related mechanisms in a much larger population of similar environmental exposure.

It is considered unethical to obtain lung biopsy from healthy human subjects; hence analysis of saliva, sputum, and BAL fluid is usually used in research as alternative approaches to study microbiome in the lung. However, these samples from alternative locations may contain possible contamination from the upper respiratory tract (Man et al., 2017). Therefore, direct analysis of lung tissue can provide a more accurate assessment of the microbiome in lung cancer. The strength of this study is that lung tumor tissues were matched with adjacent normal tissues from the same patient. This type of sampling will automatically facilitate matching for smoking/tobacco products, medications, and genetic background, just to name a few.

The chronic activation of the innate immune cells at sites exposed to specific microbial communities or their products can help enhance tumor development. Chronic inflammation can contribute to carcinogenesis through induction of genomic instability, alterations in epigenetic events and subsequent aberrant gene expression, enhanced proliferation of initiated cells and resistance to apoptosis (due to smoking). We proposed that the aberrant CD36 expression in LUAD or the activation of the pro-inflammatory molecules like the cytokines or chemokines (i.e., IL-4) can turn on the angiogenic switches mainly controlled by vascular endothelial growth factor (EGF), thereby inducing inflammatory angiogenesis and tumor cell-stroma communication (Figure 8). This, together with the changes in microenvironment due to the influence of competing microbial communities other than *Cyanobacteria*, may activate other host biological pathways important in cancer development.

Repeated exposure to microbial infection and cigarette smoke can impair the ability of macrophages to ingest apoptotic cells (Cosio et al., 2009). It is likely that if the patients are smokers, particulate matter or microbes in tobacco can get inhaled deep into the lung alveoli where they are recognized by macrophages or CD36 receptors (Figure 8). The alveoli harbor Type I epithelial cells, which act as junctions for different inflammatory molecules. The macrophages arise from the blood monocytes, then migrate into the lung where they undergo differentiation and maturation. Typically, macrophages will phagocytose foreign particles leading to diverse pro-inflammatory mediators (e.g., TNFs and ILs) (Figure 8). Macrophages have TLRs that recognize diverse microbes and toxins (Akira et al., 2006). Some of the receptors of acute inflammation include the host *CD36* (Savill et al., 2002; Gantner et al., 2003). *CD36* participates in macrophage internalization of a variety of particles and has been implicated in inflammatory responses to many of these ligands (Figure 8). *CD36* mediates internalization of particles, including microorganisms, independently of TLR signaling, but can functionally cooperate with TLRs to enhance internalization (Erdman et al., 2009). Aberrant expression of *CD36* may prevent these processes from taking effect. The aberrant CD36 expression in our samples may be due to microcystin from the *Cyanobacteria*, which increases the expression of ribosomal protein S27A (RPS27A). Pathway analysis revealed an increased abundance of sequence reads for ribosome pathway in the LUAD samples confirming the role of RPS27A (Figures 4B, 7, 7A). Increased expression of RPS27A has been shown to inhibit CD36 expression. This process may enhance the inflammation and lead to cancer.

To identify possible microcystin-gene interactions and their interactions with the innate immune system through *CD36*, we used the curated Comparative Toxicogenomic Database. This database helps identify pre-disease biomarkers resulting from environmental exposures, such as microcystin. Two transcription factors (*SREBF1and HNF4A)* and a ribosomal protein that could possibly explain the suppressed expression of CD36 in the LUAD patient samples (Figure 7A). Prolonged sublethal microcystin exposure decreased the expression of *SREBF1* mRNA in mice, which further decreased the expression of *CD36* in thyroid dysfunction and metabolic disorders in mice (Clark et al., 2007). *HNF4A* mRNA reduced CD36 expression (Zhao et al., 2015), which increases the expression of RPS27A, which then inhibits the expression of CD36 (Cai et al., 2016). These predicted observations were validated in our LUAD patient tissues, Figures 7B,C.

Interestingly, our pathway analysis predicted that presence of microcystin initiates an inflammatory process from which pro-inflammatory cytokines: TNF, type alpha (TNFα), interleukin 1-beta (IL-1β), oncostatin M (OSM), and interleukin-4 (IL-4). These pro-inflammatory factors interact with other cells of the lung, and the response of these cells is thought to accelerate, amplify, and prolong pulmonary inflammation. Along these lines, the innate immune cells are known to sense pathogen-associated molecular patterns such as viral RNAs and bacteria to produce type I interferons and proinflammatory cytokines. This response is critical in the defense against viral or bacterial infection. Excessive sensing and overwhelming host cytokine production can lead to tissue damage and autoimmune disease. Interestingly, TRIM29 was identified as a key negative regulator of the production of type I interferons as well as proinflammatory cytokines in the lungs (Xing et al., 2016). Suppressing TRIM29 expression has been shown to lead to an increased innate inflammatory response. Assessing if continuous stimulation of the lung tissues with *Cyanobacteria* and/or microcystin inhibits TRIM29 process may have important implications for the understanding of innate immunity and pathogenesis of lung cancer.

The *Cyanobacteria* population dynamics and the mechanisms regulating microcystin production remain elusive, both physiologically and ecologically. It has been reported that that nitrogen (N) speciation and inorganic carbon (C) availability might be important drivers of population dynamics *Cyanobacteria* and that an imbalance in cellular C:N ratios may trigger microcystin production. Precipitous declines in ammonium concentrations lead to a transitional period of N stress, while increases may down-regulate microcystin synthesis. Similarly, high C:N ratios are strongly correlated to the toxic phase; hence, it is likely that C and N metabolism may regulate microcystin production physiologically and ecologically. We hypothesize that an imbalance between 2-oxoglutarate and ammonium in the cell regulates microcystin synthesis in the environment. The heatmap in Figure 4D reveals abundance of *Cyanobacteria* sequences associated with D-Glutamine and D-glutamate metabolism. This is likely due to microenvironmental stress introduced by competitive microbial communities that destabilize the C:N ratios and favor the colonization of *Cyanobacteria* and production of the microcystin in LUAD, triggering the inflammatory processes.

The analysis of inflammatory molecules and microcystin identified in this study was done in FFPE samples and was validated in lung cancer cell line and LUAD fresh frozen samples harboring *Cyanobacteria*. This finding would be important in understanding the role of microcystin in inflammation and carcinogenesis. Whether microcystin is released by the inhaled live or dead *Cyanobacteria* or if microcystin is inhaled directly from the untreated surface waters and the environment was not specifically analyzed in this paper but is worth looking into. This study warrants another validation using larger sample size and population of similar environmental exposure.

In summary, this article highlights the probable role of various pro-inflammatory mediators in carcinogenesis and their promise as potential targets for chemoprevention of inflammation-associated carcinogenesis. These results provide an initial estimate of secondary metabolite gene expression, functional partitioning and functional interplay in lung-specific microenvironments. The pathways identified included reported pathways that are important in innate inflammatory roles that potentially may lead to lung cancer. The results suggest that activities of the identified pathways are necessary for competitive dominance in the lung cancer microenvironment. The dominant source of microbiota sequence reads from *Cyanobacteria* implies that specific genes that code for the microcystin toxin may be present. Taken together, this study is a cross-sectional (i.e., snapshot) study that acquired lung tumor samples from the tissue biorepository retrospectively. Like any cross-sectional study, we identified the distribution of microbiota and their product at that particular point in time given the NSCLC phenotypes. The identification of the different types of microbes, for example, presence of *Cyanobacteria* in LUAD should be studied further in a prospective format in future studies with more stringent sample collection and handling to give further insight into microbial patterns in lung cancer and at different stages of the disease. Additional longitudinal studies with larger sample sizes are essential to investigate the mechanistic links between the microbiome and lung cancer.

## Declarations

We believe that the present study has been performed in accordance with the principles and ethical guidelines for epidemiological research.

## Availability of data and material

All data generated or analyzed during this study are included in this published article [and its supplementary information files]. The datasets generated during and/or analyzed during the current study are available from the corresponding author on reasonable request. The raw data is submitted to SRA database SRP148741.

## Ethics statement

Fresh frozen lung tissue samples were obtained from UAMS tissue bank following IRB protocol approved by UAMS institutional review board [protocol # 202880].

## Author contributions

All authors of this research paper have directly participated in the planning, execution, or analysis of the study. MO: Conception and design; JJ, EB, GB, RP, BG, PJ, PP, TW, DU, MO: Analyses and interpretation; JJ, PF, EB, RP, MO, GB, NS, KA, MS, IN, DU: Drafting and writing manuscript for intellectual content; RP, LA, PA: RNA and Protein extraction and quantitation; MO, NS, GB, SJ, KA, and MS: Phenotyping and clinical significance; MO: Coordination of experiments and analyses; All authors read and approved the final manuscript.

### Conflict of interest statement

The authors declare that the research was conducted in the absence of any commercial or financial relationships that could be construed as a potential conflict of interest.
