# Supplementary material for: PARP1 Is Up-Regulated in Non-small Cell Lung Cancer Tissues in the Presence of the Cyanobacterial Toxin Microcystin
Source: Front Microbiol. 2018 Aug 6;9:1757. doi: 10.3389/fmicb.2018.01757 (PMC6087756; doi:10.3389/fmicb.2018.01757)
Supplement: Supplementary file 1 [file Data_Sheet_1.pdf]

PARP1 is up-regulated in Non-small cell lung cancer tissues in the presence of the cyanobacterial toxin microcystin

Patrick L. Apopa[1], Lisa Alley[1], Rosalind B. Penney[2], Konstantinos Arnaoutakis[3], Mathew A. Steliga[3], Susan Jeffus[4], Emine Bircan[1], Banu Gopalan[5], Jing Jin[1], Preecha Patumcharoenpol[6], Piroon Jenjaroenpun[6], Thidathip Wongsurawat[6], Nishi Shah[7], Gunnar Boysen[6], David Ussery[6], Intawat Nookaew[6], Pebbles Fagan[8], Gurkan Bebek[9,10,11]*, and Mohammed S. Orloff[1, 3]*

[1]Department of Epidemiology, University of Arkansas for Medical Sciences, Little Rock, Arkansas, United States of America
[2]Department of Environmental and Occupational Health, University of Arkansas for Medical Sciences, Little Rock, Arkansas, United States of America
[3]Winthrop P. Rockefeller Cancer Institute, University of Arkansas for Medical Sciences, Little Rock, Arkansas, United States of America
[4]Department of Pathology, University of Arkansas for Medical Sciences, Little Rock, Arkansas, United States of America
[5]Cleveland Clinic, Cleveland, Ohio, United States of America
[6]Department of Biomedical Informatics, University of Arkansas for Medical Sciences, Little Rock, Arkansas, United States of America
[7]College of Medicine, University of Arkansas for Medical Sciences, Little Rock, Arkansas, United States of America
[8] Department of Health Behavior and Health, University of Arkansas for Medical Sciences, Little Rock, Arkansas, United States of America
[9] Department of Electrical Engineering and Computer Science, Case Western Reserve University, Cleveland, Ohio, United States of America
[10]Center for Proteomics and Bioinformatics, Case Western Reserve University, Cleveland, Ohio, United States of America
[11]Department of Nutrition, Case Western Reserve University, Cleveland, Ohio, United States of America

Running Head: Cyanobacteria and microcystin increases PARP1 in NSCLC
Number of words: 4,742
Number of figures: 7

*** Corresponding Authors:**
Mohammed S. Orloff, PhD, Department of Epidemiology
Fay W. Boozman College of Public Health, University of Arkansas for Medical Sciences
4301 W. Markham Street, # 820. Little Rock, AR 72205
Phone: 501-526-6668 (Office)
Email: MSOrloff@uams.edu

Gürkan Bebek, Ph.D., Center for Proteomics and Bioinformatics, Department of Nutrition
Case Western Reserve University, 10900 Euclid Ave. BRB 921, Cleveland, Ohio 44106-4988
Phone: 216-368-4541
Email: gurkan.bebek@case.edu

Conflict of Interest
The authors declare that they have no conflicts of interest to disclose.

Supplementary Materials

The best alignments or homology hits were at 92% with uncultured *Cyanobacteria*: GenBank: GQ502588.1, FJ024312.1, and KU667126.1 and KM892905.1. All these sequences link to the *Taxonomy ID:* 1211 (NCBI:txid1211)

Lineage (full):
cellular organisms; Bacteria; *Terrabacteria* group; *Cyanobacteria/Melainabacteria* group; *Cyanobacteria*; environmental samples

Based on our OTU identifications we have investigated the sequence similarity of *Cyanobacteria* specific sequences or OTUs. These sequences are:

>8F21

CCTACGGGAGGCAGCAGTGGGGAATTTTCCGCAATGGGCGAAAGCCTGACGGAGCAATGCCGCGTGGAGGTAGAAGGCCCACGGGT
CGTGAACTTCTTTTCTCGGAGAAGAAACAATGACGGTATCTGAGGAATAAGCATCGGCTAACTCTGTGCCAGCAGCCGCGGTAAGAC
AGAGGATGCAAGCGTTATCCGGAATGATTGGGCGTAAAGCGTCTGTAGGTGGCTTTTCAAGTCCGCCGTCAAATCCCAGGGCTCAAC
CCTGGACAGGCGGTGGAAACTACCAAGCTGGAGTACGGTAGGGGCAGAGGGAATTTCCGGTGGAGCGGTGAAATGCTTAGATATTA
CATGGAATACCAATTGCGAAGGCAGGTTACTATGAGTTAATTGACGCTGATGGACGAAAGCGTGGGTAGCGAACAGGATTAGATAC
CCTGGTAGTC

>53CC

CCTACGGGAGGCAGCAGTGGGGAATTTTCCGCAATGGGCGAAAGCCTGACGGAGCAATGCCGCGTGAAGGCAGAAGGCCCACGGGT
CATGAACTTCTTTTCTCGGAGAAGAAAAAATGACGGTATCTGAGGAATAAGCATCGGCTAACTCTGTGCCAGCAGCCGCGGTAAGAC
AGAGGATGCAAGCGTTATCCGGAATGATTGGGCGTAAAGCGTCTGTAGGTGGCTTTTCAAGTCCGCCGTCAAATCCCCGGGCTCAAC
CCTGGACAGGCAGTGGAAACTACCAAGCTGGAGTACGGTAGGGGCAGAGGGAATTTCCGGTGGAGCGGTGAAATGCGTTGAGATCG
GAAAGAACACCAACGGCGAAAGCACTCTGCTGGGCCGACACTGACACTGAGAGACGAAAGCTAGGGGAGCAAATGGGATTAGATA
CCCTGGTAGTC

Further, we have conducted multi sequence alignment involving our sequences with GQ502588.1, FJ024312.1, KU667126.1 and KM892905.1. Then we drew tree to show sequences relationship using of the distances corrected by Kimura's 2 parameter model (1). Neighbor-joining Method was also used to reconstruct phylogenetic tree (2).

Cladogram

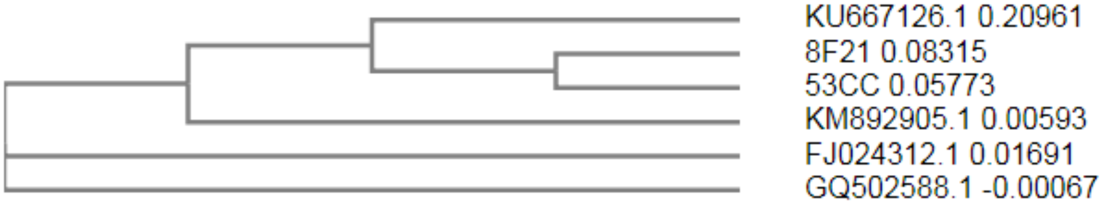

The sequences seem to share a common ancestor, but KU667126.1, 8F21, 53CC and KM892905.1 are much more closely related.

Supplementary Figures, Tables and Legends:

Figure S1: Bacterial DNA amplification: The PCR product of genomic DNA was used as a negative control. Lane 1 shows size marker, 1-kb ladder, lane 2 contain genomic DNA, lanes 3- 12 contain FFPE microbial DNA from different patient samples, lane 13 contains FFPE genomic DNA and lane 14 is blank

negative control. 500 bp fragment was amplified by the 16S primer.

There was no such fragment in total genomic DNA or total FFPE DNA.

Figure S2. Bacterial DNA amplification using primers specific for Cyanobacteria with indicated percentage abundance. Primers CYA106F and CYA781R(a) (3). Samples 9386-S4-LUAD amplified at the correct size and sample 9378-S3-LUAD showed a much lower amplification amounts proportional to amounts observed via MiSeq. A blank negative control and total human genomic DNA showed no amplification of *Cyanobacteria*.

Figure S3. Screening for unwanted microbial contamination in random FFPE samples. PCR amplification of FFPE extracted samples using 16S rRNA specific primers. Lanes 1 and 2 contain 500bp PCR products from FFPE tissue samples. Lanes 3 and 4 contain nothing or diminishing amounts of PCR products from adjacent paraffin samples. Lane 5 which was negative control (filtered and sterilized water), did not show any PCR product.

Table S1: The probes that are annotated with CD36 and the significance of differential expression between the healthy and LUAD samples in GSE19188 (4) is shown. Differentially expressed genes were estimated by using empirical Bayes framework after data was quantile normalized.

Table S2: The number of sequences generated from each sample are shown in a table. The values ranged from 32,846 to1,461,977 sequences.

Supplementary Figures

Figure S1:

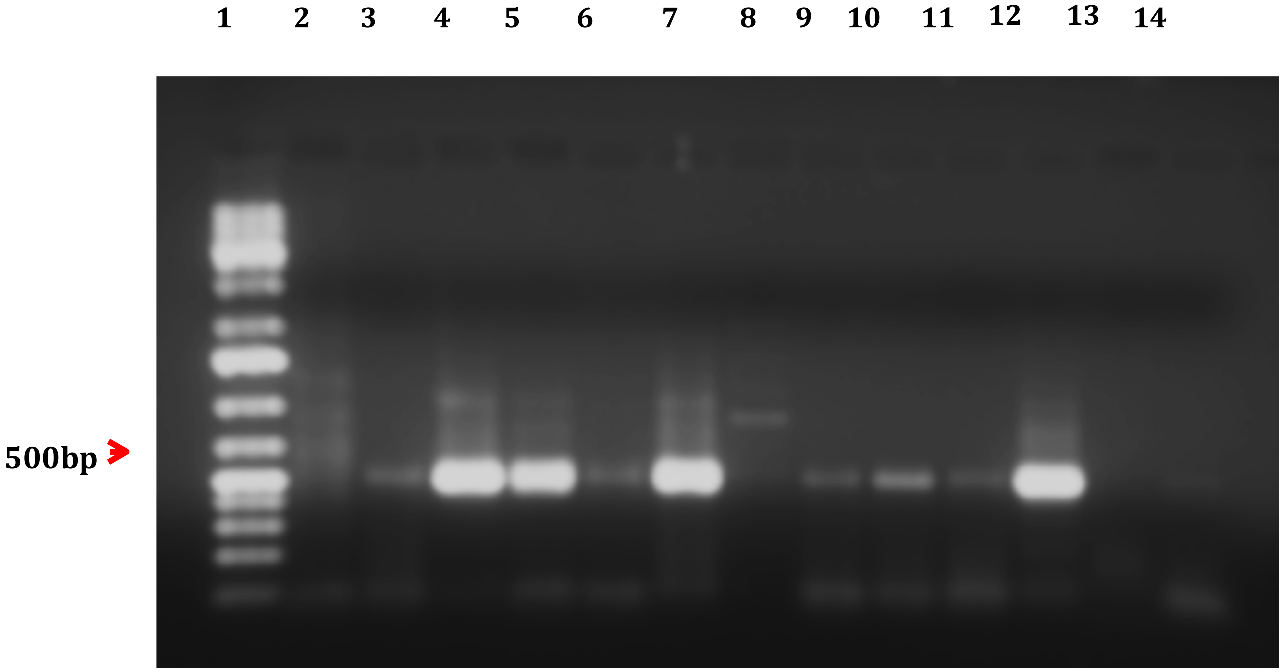

Figure S2:

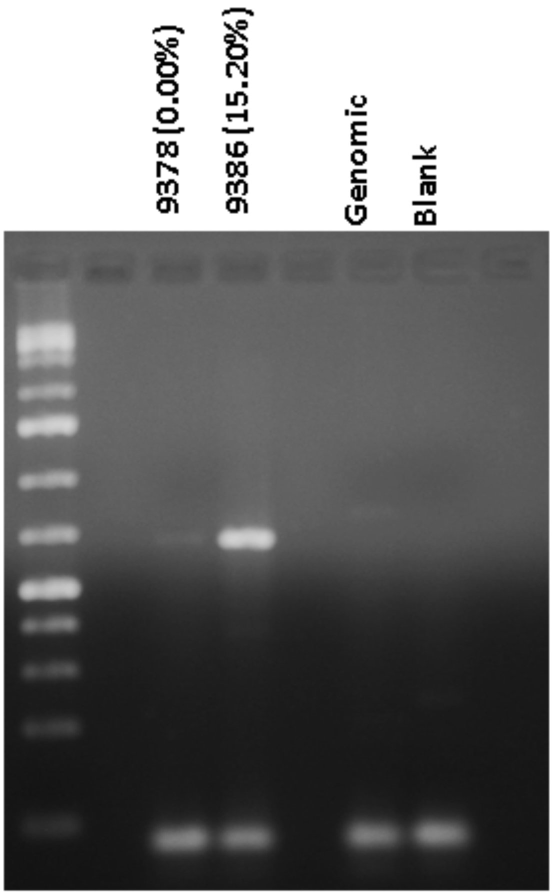

Figure S3

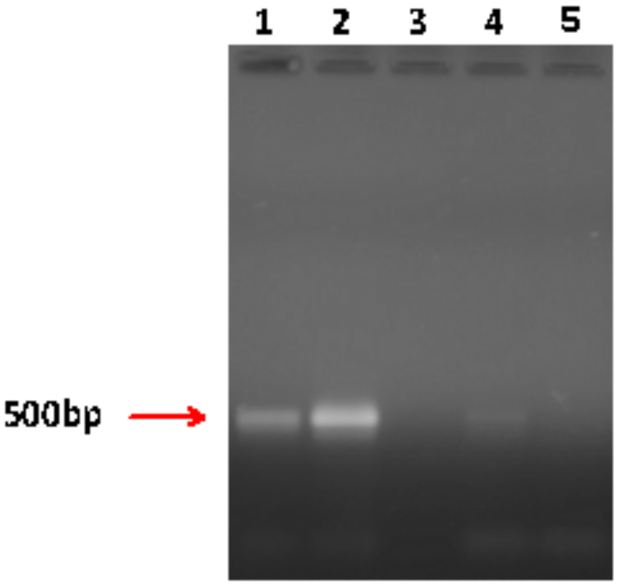

Supplementary Tables

Table S1

**Gene Symbol**	**Probe ID**	**P-value**	**FDR Adj. p-value**
CD36	228766_at	2.52E-27	1.00E-24
CD36	206488_s_at	1.13E-22	1.63E-20
CD36	228766_at	2.52E-27	1.00E-24
CD36	206488_s_at	1.13E-22	1.63E-20
CD36	209555_s_at	2.25E-20	2.05E-18
CD36	242197_x_at	3.96E-07	2.91E-06

Table S2

Sample ID	Sequence count
538E-S12-LUSC	539497
53CD-S6-NORM	734616
53D0-S2-LUAD	493261
53D1-S4-NORM	449415
53D8-S24-NORM	759613
542B-S26-LUSC	625826
542C-S3-NORM	409201
5439-S17-NORM	662371
560F-S25-LUSC	389137
5610-S7-NORM	671010
562A-S10-LUSC	700965
562B-S20-NORM	538137
8CDD-S23-LUSC	555181
8D34-S19-LUAD	403607
8DC0-S16-LUSC	32846
8DE8-S15-LUAD	622869
8E9B-S13-LUSC	538947
8F21-S1-LUAD	792561
8F46-S8-LUAD	403899
8F4C-S11-LUAD	325776
8FBC-S22-NORM	376177
9374-S21-LUAD	343436

Reference:

1. Kimura M. A simple method for estimating evolutionary rates of base substitutions through comparative studies of nucleotide sequences. *J Mol Evol* (1980) 16(2):111-20. PubMed PMID: 7463489.
2. Saitou N, Nei M. The neighbor-joining method: a new method for reconstructing phylogenetic trees. *Mol Biol Evol* (1987) 4(4):406-25. doi: 10.1093/oxfordjournals.molbev.a040454. PubMed PMID: 3447015.
3. Nubel U, Garcia-Pichel F, Muyzer G. PCR primers to amplify 16S rRNA genes from cyanobacteria. *Appl Environ Microbiol* (1997) 63(8):3327-32. PubMed PMID: 9251225; PubMed Central PMCID: PMCPMC168636.
4. Hou J, Aerts J, den Hamer B, van Ijcken W, den Bakker M, Riegman P, et al. Gene expression-based classification of non-small cell lung carcinomas and survival prediction. *PLoS One* (2010) 5(4):e10312. doi: 10.1371/journal.pone.0010312. PubMed PMID: 20421987; PubMed Central PMCID: PMCPMC2858668.

1.
